# Limitations of laparoscopy to assess the peritoneal cancer index and eligibility for cytoreductive surgery with HIPEC in peritoneal metastasis

**DOI:** 10.1007/s00423-022-02455-2

**Published:** 2022-02-02

**Authors:** Can Yurttas, Lisa Überrück, Giorgi Nadiradze, Alfred Königsrainer, Philipp Horvath

**Affiliations:** 1 Department of General, Visceral and Transplant Surgery, University Hospital Tübingen, Hoppe-Seyler-Str. 3, 72076 Tübingen, Germany; 2 Department of Gastroenterology, Gastrointestinal Oncology, Infectious Disease and Geriatric Medicine, University Hospital Tübingen, Otfried-Müller-Str. 8, 72076 Tübingen, Germany

**Keywords:** Peritoneal carcinomatosis, Peritoneal cancer, Peritonectomy, PCI, Hyperthermia

## Abstract

**Purpose:**

We aimed to determine the value of laparoscopy to assess the intra-abdominal tumor extent and predict complete cytoreduction.

**Methods:**

All patients at our department in the period from 2017 to 2021 that underwent laparoscopy to assess peritoneal metastasis and subsequent open exploration with the intention to perform cytoreductive surgery (CRS) with HIPEC were retrospectively identified in a continuously maintained database.

**Results:**

Forty-three patients were analyzed. Peritoneal cancer index (PCI) determination by laparoscopy compared to open surgery was overestimated in five patients (11.6%), identical in eleven patients (25.6%), and underestimated in 27 patients (62.8%). PCI differences were independent of surgeons, tumor entities, and prior chemotherapy. Thirty-four patients (79.1%) were determined eligible for CRS with HIPEC during open exploration, whereas nine patients (20.9%) underwent a non-therapeutic laparotomy. Complete or almost complete cytoreduction was achieved in 33 patients (76.7%). In one patient, completeness of cytoreduction was not documented.

**Conclusions:**

We demonstrate a moderate agreement according to weighted Cohen’s kappa analysis of PCI values calculated during laparoscopy and subsequent open exploration for CRS with HIPEC. Uncertainty of PCI assessment should therefore be kept in mind when performing laparoscopy in patients with peritoneal metastasis.

## Introduction

Peritoneal metastasis (PM) comprises primary tumors of the peritoneum as well as peritoneal seedings from other solid tumor sites. Numerous tumor entities, such as colorectal, gastric, and ovarian carcinoma, can cause peritoneal spread of tumor cells. PM used to be seen as the final stage of the disease and was therefore treated with palliative intention only. Meanwhile, the technique of cytoreductive surgery (CRS) and hyperthermic intraperitoneal chemotherapy (HIPEC) has been established for the treatment of selected patients with PM. This combined treatment includes peritonectomy and visceral organ resection aiming to completely remove abdominal and pelvic tumor formations. Subsequently, HIPEC is performed to eliminate remaining tumor and free peritoneal tumor cells. For certain entities, such as pseudomyxoma peritonei and malignant peritoneal mesothelioma, combination therapy of CRS and HIPEC became the standard of care [1, 2]. Many authors also advocated this form of therapy as a standard of care for PM of the ovary [3], colorectal tumors and tumors of the appendix [1, 4, 5]. The preconditions for this form of therapy are the absence of extra-abdominal metastases, limited PM depending on the tumor entity, and macroscopically complete tumor removal. To determine the extent of PM during open exploration of the abdominal cavity, the assessment of the peritoneal cancer index (PCI) according to Jacquet et al. [6] has been established. In general, the higher the grading or aggressiveness of the disease, the lower the PCI value should be for suitability for complete macroscopic CRS and HIPEC. For example, up to a PCI value of 17, a cytoreductive approach with HIPEC for peritoneal metastatic colorectal carcinoma appears to be reasonable from an oncological point of view [7]. For PM of ovarian origin no PCI-threshold has been defined so far [8–10].

Diagnostic laparoscopy has become a widely used tool to estimate the extent and distribution within the abdominal cavity of PM during staging process in order to assess the potential oncologic effectiveness of CRS with HIPEC.

In the recent literature, several reports exist on the use of diagnostic laparoscopy to assess intra-abdominal tumor burden in order to reduce the proportion of nontherapeutic laparotomies [11–17]. These nontherapeutic laparotomies still affect approximately 40% of all patients today [15, 17] and are not only a limitation of quality of life for the patients, but are also associated with a significant postoperative complication rate, delay of further therapy, and relevant costs due to the logistic efforts for planned CRS and HIPEC.

Compared with radiological imaging techniques such as computed tomography (CT), diagnostic laparoscopy as a surgical procedure is associated with a greater time and organizational effort, potential complications such as mainly access injuries of abdominal organs due to previous surgical interventions, and the side effects of anesthesia [18]. In contrast, imaging techniques are well suited to detect extraperitoneal tumor manifestations, whereas CT morphologic abnormalities of the peritoneum show varying correlation with PCI values obtained during surgical exploration [12, 19].

Despite the described advantages of laparoscopy and the low complication rate, nontherapeutic laparotomies still occur after previous laparoscopic evaluation. Therefore, we aimed to determine the significance of laparoscopic assessment of intra-abdominal tumor extent in our patient population and identify delay to CRS with HIPEC due to previously performed laparoscopy.

## Materials and methods

### Trial design and data collection

Patients in the period from 2017–2021 with suspected PM who underwent laparoscopy to define the PCI at the Department of General, Visceral and Transplant Surgery Tübingen, Germany, documented in a continuously maintained data base were identified. Patients that underwent laparoscopy with consecutive open exploration with the intention to perform cytoreductive surgery with HIPEC were selected and included in the analysis, whereas chemotherapy treatment between laparoscopy and open exploration was an exclusion criterion. Patients that had completed neoadjuvant chemotherapy prior to laparoscopic PCI assessment were included. Data comprised age, sex, date of primary diagnosis and peritoneal metastasis, histology of primary tumor and PM, prior chemotherapy, CT-confirmed findings of PM, date of laparoscopy and open exploration, PCI values during laparoscopic and open assessment. No CT-based PCI calculations were performed. Detailed information of laparoscopy including incision to suture time, number of trocars in use and surgeon in charge as well as of open surgery exploration with possibility and extent of complete cytoreduction were collected and analyzed. All included patients underwent complete staging workup and were discussed in a multidisciplinary tumorboard. The Ethics Committee of the University Hospital of Tübingen approved this study and it was registered with project identifier *034/2021BO2*.

### Laparoscopy

Laparoscopy was performed or supervised by consultants. A 30° camera (KARL STORZ SE & Co. KG) was introduced under general anesthesia through an optic trocar after establishment of capnoperitoneum by Veress needle. Depending on the intraabdominal situs and surgeon’s estimation up to two additional trocars were inserted under vision. The reasons for the number of trocars used for the assessment were not specifically defined in the operative reports. Intraabdominal adhesions were cleared if necessary for unrestricted sight. Hence, the abdominal cavity with all 13 abdominopelvic areas was systematically inspected, PCI was calculated according to Jacquet et al. [6] and the possibility to perform cytoreductive surgery with complete cytoreduction evaluated. The lesser sac was opened and examined if deemed necessary by the consultant in charge. Samples for histopathological examination were taken from suspicious nodules exemplarily and peritoneal washing cytology was obtained according to surgeon’s estimation. Each region was scored individually and the final calculation was performed immediately after surgery with both the score for each region and the final calculation provided in the operative report. All patients were discharged after full postoperative recovery.

### Cytoreductive Surgery with HIPEC

If patients were deemed suitable for CRS with HIPEC usually a new admission for surgery was arranged. Following midline laparotomy, the abdominal cavity was systematically examined for peritoneal tumor deposits, PCI was re-assessed in the above described manner and possibility of complete cytoreduction investigated. Reassessment was not necessarily performed by the same surgeon that evaluated PCI during laparoscopy. CRS included procedures of parietal peritonectomy, visceral organ resection and thermic destruction of peritoneal tumors. If complete (CC-0) or almost complete cytoreduction (CC-1) could be achieved, decision for HIPEC was made. HIPEC was administered and performed in a closed abdominal technique utilizing a roller pump (Performer HT, RanD Biotech, Medolla, Italy). The abdominal cavity was temporarily closed using a running suture, filled up with either 0.9% saline in case of cisplatin or mitomycin c or dextrose when oxaliplatin was used, heated to 42.0 °C before application of chemotherapeutic agents and conducted for 60 min. Patients with PM deriving from colorectal, appendiceal, cancer of unknown primary and tumors of the small intestine were treated with 100 mg/m^2^ cisplatin, whereas PM from gastric cancer, carcinoma of the esophagogastric junction and mesothelioma were exposed to 75 mg/m^2^ cisplatin in combination with 15 mg/m^2^ doxorubicin. Body surface area was calculated with the formula of Dubois and Dubois [20]. Afterwards, the abdomen was rinsed and once more explored before definite abdominal wall closure.

### Outcome measures and statistical analysis

The primary outcome measure was the discrepancy of PCI-values detected during laparoscopic and open surgery assessment. Secondary evaluations were the number of patients that were deemed not-resectable during open exploration, time to CRS because of prior laparoscopic exploration and complications that arose in context with laparoscopy. Time from laparoscopy to open exploration was considered as duration of delay to open exploration with intention to perform CRS with HIPEC. Positive predictive value was calculated as the number of patients that underwent CRS with achieved CC-0 and CC-1-status respectively among all patients assessed for eligibility by laparoscopy. Microsoft Excel 2019 (Microsoft Corporation) was used for data maintenance. SPSS Statistics Version 28 (IBM) was used for calculation of Cohen’s Weighted Kappa [21]. Design of figures was performed with GraphPad PRISM 9 (Graphpad Software, Inc.).

## Results

### Patient characteristics, screening, and study enrollment

We retrospectively identified 19 women (44.2%) and 24 men (55.8%) between the age of 27 and 83 (mean 56.0 years) in a continuously maintained database that first underwent laparoscopic exploration for suspected PM and were assessed eligible for complete cytoreduction. Patients that received chemotherapy between laparoscopy and open exploration were excluded. All 43 included patients were subjected to exploratory laparotomy with the intention to perform CRS with HIPEC.

### Details of tumor type, extent, and concomitant therapy

Of 43 patients with PM included, in nine patients (20.9%) tumor derived from the appendix vermiformis with histological detection of low grade appendiceal mucinous neoplasm (LAMN) in eight patients (18.6%) and goblet cell carcinoid in one patient (2.3%). The origin of PM was ascribed to colorectal cancer in nine patients (20.9%), to the small intestine in one patient (2.3%) and was not identifiable in one patient (2.3%) who was further treated as cancer of unknown primary (CUP). Four patients (9.3%) suffered from peritoneal mesothelioma, one from urachal duct carcinoma (2.3%) and one (2.3%) from uterine leiomyomatosis. Most patients included had PM from gastric cancer (n = 17, 39.5%) of which five tumors (11.6%) were located at the esophagogastric junction.

**Fig. 1:**
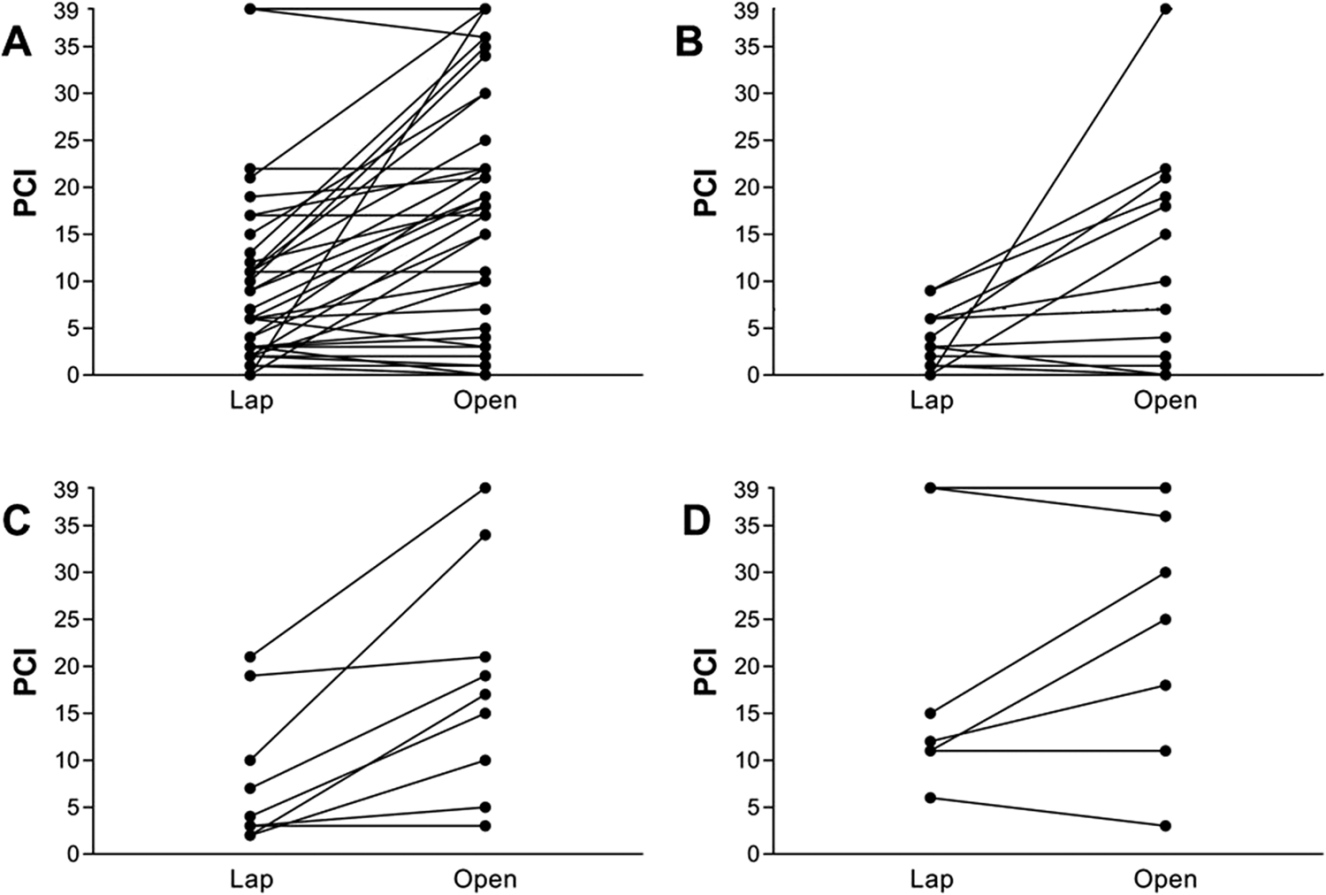
(A) PCI differences of laparoscopy compared to open exploration in all patients, (B) patients with gastric cancer, with (C) colorectal and (D) appendiceal neoplasms, *lap* laparoscopy, *PCI* peritoneal cancer index

All but seven patients (16.3%) were diagnosed with simultaneous occurrence of PM. Twenty-seven patients (62.8%) received chemotherapy before laparoscopic evaluation of intraperitoneal tumor extent. Detailed information about patient and tumor characteristics are further described in Tables 1 and 2, Fig. 1.

**Table 1:** Characteristics of patients with peritoneal metastasis that underwent laparoscopy and subsequent open exploration for intended cytoreductive surgery with HIPEC between 2017 and 2020. *CUP* cancer of unknown primary

Patient characteristics	
Ethnicity (***n*** = (%))	
Caucasian	43 (100)
Age in years (mean (range))	56.0 (27–83)
Sex (***n*** = (%))	
Female	19 (44.2)
Male	24 (55.8)
Tumor characteristics	
Primary tumor (*n* = (%))	
Esophagogastric junction and gastric carcinoma	17 (39.5)
Colorectal cancer	9 (20.9)
Appendiceal neoplasia	9 (20.9)
Mesothelioma	4 (9.3)
Small intestine	1 (2.3)
Urachal duct carcinoma	1 (2.3)
Cancer of unknown primary (CUP)	1 (2.3)
Uterine leiomyoma	1 (2.3)
Onset of peritoneal metastasis (*n* = (%))	
Synchronous	36 (83.7)
Metachronous	7 (16.3)
Previous systemic chemotherapy (*n* = (%))	
Yes	27 (62.8)
No	16 (37.2)

**Table 2:** Findings during laparoscopic assessment of peritoneal cancer index according to tumor entity and difference to PCI values evaluated during subsequent open exploration. *CUP* cancer of unknown primary *PCI* peritoneal cancer index

	Laparoscopic Surgery	Open Surgery
PCI (mean (range))		
All	10.2 (0–39)	17.3 (0–39)
Esophagogastric junction and gastric carcinoma	3.2 (0–9)	9.3 (0–39)
Colorectal cancer	7.9 (2–21)	18.1 (3–39)
Appendiceal neoplasia	23.4 (6–39)	26.7 (3–39)
Mesothelioma	17.3 (13–22)	24.3 (17–36)
Small intestine	2.0	1.0
Urachal duct carcinoma	11.0	30.0
Cancer of unknown primary (CUP)	9.0	22.0
Uterine leiomyoma	11.0	35.0

### PCI calculation during laparoscopic and open exploration

**Fig. 2:**
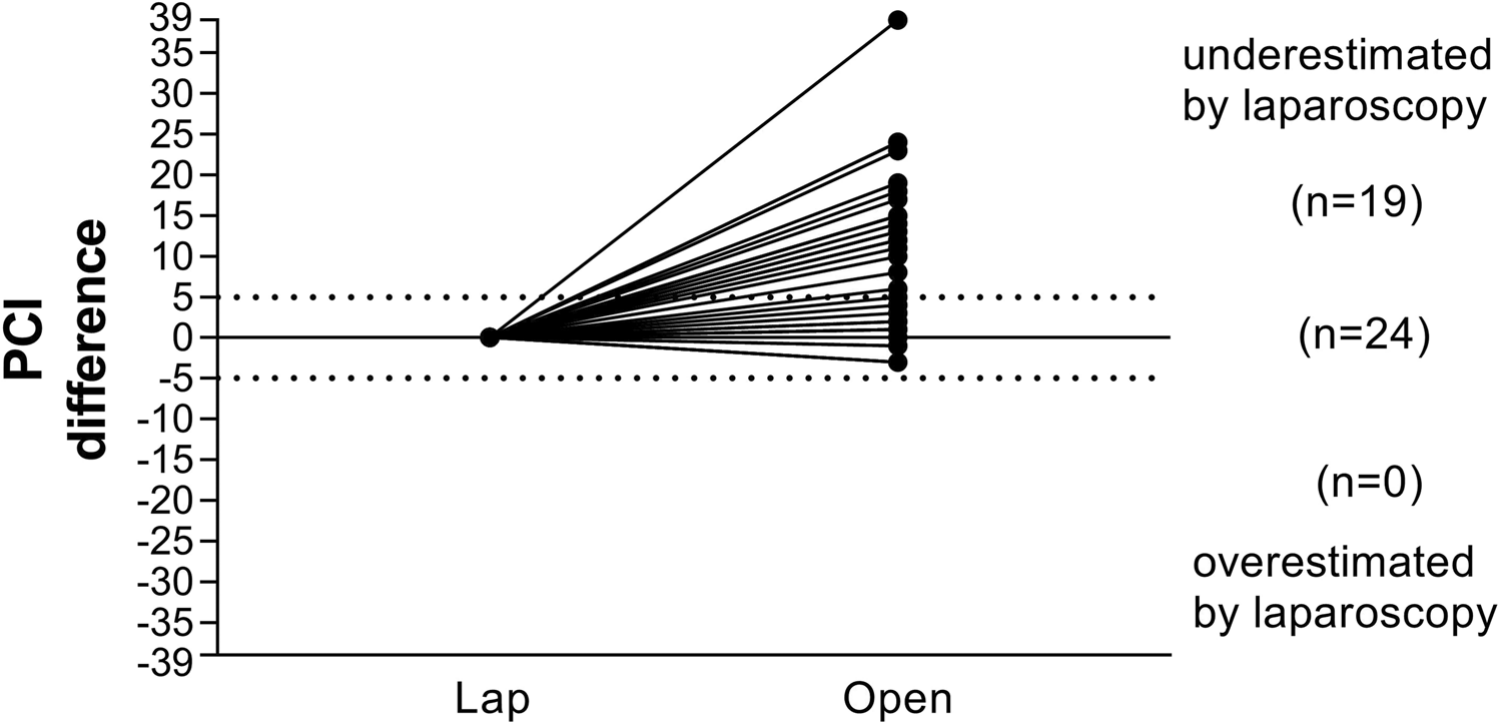
PCI differences of laparoscopic compared to open exploration in all patients. PCI-score in 19 patients was underestimated by five or more points and in none of the patients PCI was overestimated by five or more points by laparoscopic exploration. *lap* laparoscopy, *PCI* peritoneal cancer index

Laparoscopic PCI assessment was performed by surgeons that were not necessarily specialized in the treatment of PM. Duration of laparoscopy varied between eleven and 87 min with a mean duration of 42.8 min in 42 patients (97.7%). Incision-suture-time was not available for one patient (2.3%). The number of trocars used was at least one and at maximum three (Table 3). Laparoscopic PCI values were numbered with a mean of 10.2. Four patients (9.3%) were considered unaffected of PM during laparoscopy. Exploratory laparotomy revealed PCI values with a mean PCI of 17.3. There were no PM found in six patients (14.0%) of which two patients (4.7%) were assessed correctly by laparoscopy. PCI was determined lower during open exploration in five patients (11.6%). In eleven patients (25.6%), PCI values were identical. In the remaining 27 patients (62.8%) PCI values were underestimated by laparoscopy. Results are depicted in Fig. 2. Cohen’s weighted Kappa coefficient of PCI values determined during open and laparoscopic surgery was 0.479 (95% CI 0.322–0.636; *p* = 0.001). There was no difference in PCI assessment according to tumor entity (Table 2). Differences in PCI calculcation were higher the more trocars were utilized and the longer incision-suture-time of laparoscopy (see Table 3). PCI incongruities occurred likewise among all surgeons and tumor entities (see Fig. 1) and were independent of prior chemotherapy.

**Table 3:** PCI-differences of laparoscopic and open PCI-assessment of peritoneal cancer index according to number of trocars used for laparoscopy and incision-suture-time. *PCI* peritoneal cancer index

PCI-difference according to number of trocars (mean (range))	
1	3.6 (0–10)
2	8.5 (0–24)
3	8.3 (0–39)
PCI-difference according to incision suture time (mean (range))	
< 30 min	5.9 (0–18)
30–60	7.8 (0–24)
> 60 min	11.1 (0–39)

During open exploration, 34 patients (79.1%) were determined eligible for CRS with HIPEC, whereas nine patients (20.9%) underwent a non-therapeutic laparotomy. Complete cytoreduction (CC-0) was possible in 22 of 34 patients (64.7%). Nearly complete cytoreduction (CC-1) was achieved in 11 of 34 patients (32.4%). Complete or almost complete cytoreduction with HIPEC was feasible in eight of nine (88.9%) patients with appendiceal neoplasms at a maximum PCI-score of 39 (mean 25.5), in one patient with cancer of the small intestine at a PCI-score of one, in seven of nine (77.8%) patients suffering from colorectal carcinoma at a maximum PCI-score of 21 (mean 12.9), in 13 of 17 (76.5%) patients with gastric cancer or adenocarcinoma of the esophagogastric junction at a maximum PCI of 18 (mean 4.4), in three of four (75.0%) patients with mesothelioma at a maximum PCI of 22 (mean 20.3), in one patient each with urachal duct carcinoma and with uterine leimyoma at a score of 30 and 35 respectively. No CRS was possible in one patient with CUP at a PCI of 22. Completeness of cytoreduction status was not documented in one patient (2.3%) that underwent HIPEC.

Of those patients that were not eligible for complete cytoreduction, one patient suffered from mesothelioma (2.3%), four patients from gastric cancer (9.3%), two from colorectal cancer (4.7%), one from CUP (2.3%) and one from LAMN (2.3%). In eight patients (18.6%), PCI was underestimated by laparoscopy by seven, ten, 13, 17, 18, 23, 24 and 39 points respectively. In one patient with pseudomyxoma peritonei from LAMN, laparoscopy overestimated PCI at 39 whereas open exploration showed a PCI of 36. Additional information are found in Table 4.

**Table 4:** Results of open exploration with the intention to perform cytoreductive surgery with HIPEC. *CC* completeness of cytoreduction*, CUP* cancer of unknown primary, *HIPEC* hyperthermic intraperitoneal chemotherapy

Completeness of cytoreduction and addition of HIPEC (*n* = (%))	CC-0	CC-1	CC-2-3	n/a	HIPEC
All	22 (51.2)	11 (25.6)	9 (20.9)	1 (2.3)	34 (79.1)
Esophagogastric junction and gastric carcinoma	10 (23.3)	2 (4.7)	4 (9.3)	1 (2.3)	12 (27.9)
Colorectal cancer	6 (14.0)	1 (2.3)	2 (4.7)	0 (0.0)	7 (16.3)
Appendiceal neoplasia	3 (7.0)	5 (11.6)	1 (2.3)	0 (0.0)	8 (18.6)
Mesothelioma	2 (4.7)	1 (2.3)	1 (2.3)	0 (0.0)	3 (7.0)
Small intestine	1 (2.3)	0 (0.0)	0 (0.0)	0 (0.0)	1 (2.3)
Urachal duct carcinoma	1 (2.3)	0 (0.0)	0 (0.0)	0 (0.0)	1 (2.3)
Cancer of unknown primary (CUP)	0 (0.0)	0 (0.0)	1 (2.3)	0 (0.0)	0 (0.0)
Uterine leiomyoma	0 (0.0)	1 (2.3)	0 (0.0)	0 (0.0)	1 (2.3)

### Positive predictive value of laparoscopic assessment of eligibility for CRS and HIPEC

Of 43 patients assessed eligible for complete cytoreduction by CRS with HIPEC, nine patients (20.9%) underwent non-therapeutic laparotomy. These were considered irresectable due to extensive disease (PCI range from 19 to 39 (mean 29.8)) found during open exploration. In these patients, PCI was underestimated by laparoscopy. Eleven patients (25.6%) had almost complete cytoreduction (CC-1) and 22 patients (51.2%) complete cytoreduction (CC-0). Information about completeness of cytoreduction status was missing in one patient (2.3%). In total 34 patients were treated with HIPEC (79.1%). The positive predictive value of laparoscopy to estimate the feasibility of complete or almost complete cytoreduction is therefore 79.1% in this study cohort.

### Delay to open exploration with intention to perform CRS with HIPEC

Time from laparoscopic to open assessment varied between 2 and 71 days with a mean of 31.7 days. There were 13 patients in which open exploration was performed > 40 days (range 41—71 days, mean 52.5 days) after laparoscopy. Of these, seven patients received systemic chemotherapy prior to laparoscopy and open exploration therefore requiring a longer time interval from the last chemotherapy cycle until intended CRS with HIPEC for safety reasons. Four patients with pseudomyxoma peritonei and one patient with leiomyoma had no previous therapy. The maximum delay of 71 days was due to detection of SARS-CoV-2 after laparoscopy in one patient with mesothelioma and 67 days between laparoscopy and CRS with HIPEC in a 74 years old patient with pseudomyxoma peritonei from LAMN with a PCI of 39 due to time requested for consideration as desired by the patient. Two patients following neoadjuvant treatment for gastric cancer and cancer of the esophagogastric junction had a delay of 56 and 58 days respectively as they underwent reassessment by laparoscopy directly after termination of neoadjuvant chemotherapy but were scheduled for CRS with gastrectomy and HIPEC eight weeks later for safety reasons.

### Complications following laparoscopy and length of hospitalization

Duration of hospitalization for laparoscopic assessment and associated treatment ranged from one to ten nights respectively. In those patients subjected to laparoscopy for suspected PM, length of stay was one (30 patients; 69.8%) night, two (nine patients; 20.9%) or three (two patients, 4.7%) nights respectively. Two patients (4.7%) were first diagnosed with PM during laparoscopic appendectomy for suspected acute appendicitis. One patient (2.3%) was directly assigned to CRS with HIPEC two days after laparoscopic appendectomy with a total length of hospital stay of 10 nights. The other patient was discharged four days after appendectomy. Two patients (4.7%) experienced postoperative complications requiring hernia repair due to trocar hernia and ileocolic resection because of persistent peritonitis following appendectomy respectively both grade IIIb according to Clavien-Dindo classification of postoperative complications [22].

## Discussion

In this monocentric retrospective study, we sought to analyze the significance of laparoscopy to assess the PCI and thereby eligibility for complete cytoreduction with HIPEC. Forty-three patients with suspected PM from various tumor entities were included in this analysis, demonstrating a moderate agreement of PCI values calculated during laparoscopy and subsequent open exploration for CRS with HIPEC according to Cohen’s weighted Kappa [21]. This investigation shows impreciseness of laparoscopic PCI assessment and therefore questions its role to assess eligibility for CRS with HIPEC.

In the recent literature, diagnostic laparoscopy is described as a useful tool to reduce non-therapeutic laparotomies in patients with PM. In 174 patients with PM from colorectal cancer, a cohort of 124 patients was assessed for CRS and HIPEC by laparoscopy and the remaining 48 patients from a historical group of patients before introduction of laparoscopy had undergone laparotomy directly to estimate the extent of PM. Non-therapeutic laparotomies occurred in 35.4% of patients and therefore more often in the historical group than in the group explored by laparoscopy prior to laparotomy causing only 21.0% non-therapeutic laparotomies (*p* = 0.044). Postoperative complication rate ascribed to laparoscopy was 3.5% and included maximum grade II complications according to Clavien-Dindo classification [13].

Another retrospective analysis of 112 patients with PM from colorectal cancer analyzed the benefit of additional laparoscopy in the preoperative workup. Results of computed tomography had revealed 100 patients (89.3%) would have been eligible for CRS and HIPEC, whereas laparoscopic estimation excluded another 5 patients (4.5%) from non-therapeutic laparotomy. Overall, 95 patients (84.8%) were considered amenable for CRS with HIPEC. Eleven patients withdrew consent to perform CRS and HIPEC. Eighty-four patients underwent open exploration of which 14 patients were suspended from CRS and HIPEC during open exploration so that 70 patients were eventually treated by CRS and HIPEC. The period from laparoscopy to laparotomy was in median 41 days (4–323 days). The authors concluded, though not statistically significant (*p* = 0.125), laparoscopy to be clinically relevant due to avoidance of five unnecessary non-therapeutic laparotomies and recommend laparoscopy in case CT-based PCI-calculation exceeds a score of ten [12].

In another retrospective series of 31 patients with PM from various tumors, laparoscopy was feasible and accurate in identifying PM in all 31 patients (100%) and performed without occurrence of any complications. Twelve patients (38.7%) were deemed eligible for complete cytoreduction, whereas 17 patients (54.8%) were excluded from open exploration due to extensive abdominal tumor load with PCI > 20 or small bowel affection, respectively. Two patients underwent CRS and HIPEC despite a PCI of > 20. Of 12 patients actually deemed eligible for complete cytoreduction 10 patients (83.3%) were ultimately suitable for CRS with HIPEC. The positive-predictive value of laparoscopy was hence calculated to be 83.3% [11].

Before implementation of laparoscopic PCI assessment in 2017 as standard of care at our institution, non-therapeutic laparotomies occurred in 165 patients (29.3%) among a total of 564 patients with PM from various entities that had undergone open exploration with the intention to perform CRS with HIPEC between 2010 and 2017. Introduction of laparoscopy prior to open surgery therefore reduced non-therapeutic laparotomies by 8.4% from 29.3 to 20.9%.

Although the positive predictive value of laparoscopy to foretell feasibility of CRS was 79.1% and non-therapeutic laparotomies occurred in 20.9% of patients in our cohort, which is comparable to the results of recent research [11–13], the significance of laparoscopy in our cohort is questionable with a difference of mean PCI values of 7.1 and Cohen’s weighted Kappa coefficient of 0.479 (95% CI 0.322–0.636; *p* = 0.001). Indeed, PCI is mostly underestimated by laparoscopy, which is most likely responsible for a still considerable rate of unnecessary laparotomies. We considered the number of trocars used for laparoscopy as well as incision-suture time as surrogate parameters of elaborateness and therefore expected less differences in PCI values the more trocars were used and the longer incision-suture time was. However, the range of PCI values and the mean PCI differences increased with two and three trocars inserted and with longer duration of laparoscopy. This might also be due to the relatively small number of patients in each subgroup of this retrospective study. We also consider that one trocar only is insufficient to properly inspect the abdominal cavity for PM and rule out involvement ofe.g., the lesser sac or the intestinal mesentery. In the recent literature, there is no comparable investigation making a further interpretation of our findings difficult. Nevertheless, in order to reduce uncertainty of laparoscopy, we believe standards of care should at least be followed to minimize limitations. Performing laparoscopy with one trocar only and 15 min incision-suture time in a patient with PM from gastric cancer with a PCI of 7 during laparoscopy but 19 after open exploration doubtlessly does not add to this standard. Varying experience in laparoscopic PCI-assessment of surgeons performing laparoscopy at our center might be a possible cause for divergent PCI-values. If the number of interventions performed influences accuracy has not been investigated in this study and remains to be answered. Twenty-seven (62.8%) patients received chemotherapy prior to laparoscopic PCI-assessment. Response of PM to chemotherapy might have been misinterpreted as either vital tumor deposits or remnant tumor tissue leading to inaccuracy of PCI-values assessed during laparoscopy. This is underlined by recent findings of a prospective trial showing relevant differences of the PCI calculated during surgery and the PCI verified by pathological examination [23]. A potential explanation for increased PCI values during open exploration might also be seen in a mean delay of 31.7 days (range 2–71) from prior laparoscopy. Delay was longer in patients with either benign (leiomyoma) or indolent (LAMN) tumors or in patients that underwent laparoscopic PCI-assessment directly after termination of neoadjuvant chemotherapy but that were scheduled for CRS with HIPEC several weeks later for safety reasons. Although PCI-differences were independent of tumor entities in our cohort, aggressive PM might have progressed in the meantime though, which questions if such delay is justified.

Yet, the PCI-value alone is not the only decision criterion for eligibility for CRS and HIPEC. Other patient related factors such as age, comorbidities, histopathological and molecular pathology features of the tumor as well as distribution of PM within the abdominal cavity are also decisive factors to assess before consideration for CRS with HIPEC [24–28]. In this context, laparoscopy is indeed suitable to rule out tumor dissemination to irresectable structures of the abdominal cavity (e.g., excessive affection of small intestine) and to retrieve samples for histopathological and genetic investigations before further therapy planning.

Laparotomies may be associated with relevant peri- and postoperative complications and restraints of health-related quality of life which is why non-therapeutic open exploration should be avoided as far as possible. To rule out false estimations of laparoscopy and potential progress of PM until open surgery but at the same time to utilize laparoscopy with all its benefits, an improved approach might be to perform laparoscopy immediately before premediated open surgery with the intention to perform CRS with HIPEC. From an economic and organizational point of view in current health care structure however, unnecessary prearrangements especially for HIPEC and scheduling a day task in the operation program, which is canceled shortly before equals an internecine mismanagement. Although connected with overt advantages for affected patients, such a treatment strategy is not covered by the current health care policy and therefore remains ineligible in Germany.

The conclusions that can be drawn by this study are certainly limited due to the retrospective approach, the monocentric analysis, the heterogeneous treatment and the small number of patients included. Altogether, there are only results from retrospective analysis available in the literature, so that the significance of laparoscopy in the assessment of peritoneal tumor extension and therefore eligibility for CRS and HIPEC is restricted. Therapy-relevant ratings of PCI gained by laparoscopy should thus be applied with caution unless there are reliable data available obtained from prospective multi-centric trials.

## Conclusions

Laparoscopy to assess eligibility for CRS with HIPEC reduces non-therapeutic laparotomies but PCI assessment compared to open exploration is imprecise. We demonstrate uncertainty comprising mainly underestimation. Laparoscopic assessment of peritoneal metastasis should therefore be interpreted with caution.

## Data Availability

Data are available from the corresponding author upon request.
